# Optical damage limit of efficient spintronic THz emitters

**DOI:** 10.1016/j.isci.2021.103152

**Published:** 2021-09-21

**Authors:** Sandeep Kumar, Anand Nivedan, Arvind Singh, Yogesh Kumar, Purnima Malhotra, Marc Tondusson, Eric Freysz, Sunil Kumar

**Affiliations:** 1 Femtosecond Spectroscopy and Nonlinear Photonics Laboratory, Department of Physics, Indian Institute of Technology Delhi, New Delhi 110016, India; 2 Laser Science and Technology Center, Metcalfe House, Civil Lines, New Delhi 110054, India; 3 Univ. Bordeaux, CNRS, LOMA, UMR 5798, 33405 Talence, France

**Keywords:** Physics, Photonics, Engineering

## Abstract

THz pulses are generated from femtosecond pulse-excited ferromagnetic/nonmagnetic spintronic heterostructures via inverse spin Hall effect. The highest possible THz signal strength from spintronic THz emitters is limited by the optical damage threshold of the corresponding heterostructures at the excitation wavelength. For the thickness-optimized spintronic heterostructure, the THz generation efficiency does not saturate with the excitation fluence even up till the damage threshold. Bilayer (Fe, CoFeB)/(Pt, Ta)-based ferromagnetic/nonmagnetic (FM/NM) spintronic heterostructures have been studied for an optimized performance for THz generation when pumped by sub-50 fs amplified laser pulses at 800 nm. Among them, CoFeB/Pt is the best combination for an efficient THz source. The optimized FM/NM spintronic heterostructure having α-phase Ta as the nonmagnetic layer shows the highest damage threshold as compared to those with Pt, irrespective of their generation efficiency. The damage threshold of the Fe/Ta heterostructure on a quartz substrate is ∼85 GW/cm^2^.

## Highlights

• THz generation efficiency of (CoFeB,Fe)/(Pt,Ta) spintronic film heterostructures
• Determination of optical damage threshold at NIR excitation
• Mean value of the optical damage threshold is ∼60 GW/cm^2^

## Graphical abstract

**Figure:**
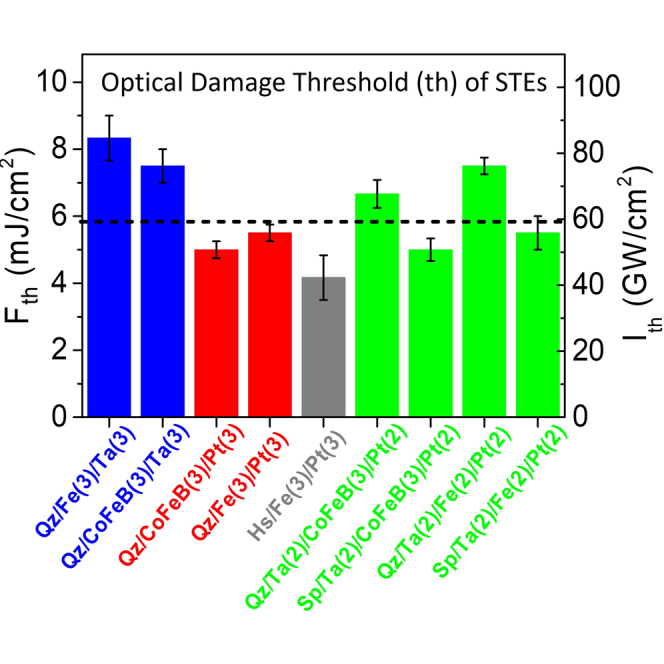

## Introduction

When ferromagnetic/nonmagnetic (FM/NM) metallic thin film heterostructures are irradiated with optical femtosecond laser pulses, emission of THz pulses takes place (Li et al., 2019; Wu et al., 2017; Seifert et al., 2016, 2017a, 2017b; Yang et al., 2016; Kampfrath et al., 2013a). Such emitters are unique in the sense that they combine the excitements from the three most active research fields, currently, the ultrafast lasers, spintronics, and THz radiation. The most commonly known process responsible for the THz pulse generation from FM/NM-type spintronic heterostructures is the inverse spin Hall effect (ISHE) (Li et al., 2019; Wu et al., 2017; Seifert et al., 2016, 2017a, 2017b; Yang et al., 2016; Kampfrath et al., 2013a). Beaurepaire et al., for the first time in 2004, observed emission of THz radiation from femtosecond laser-excited ferromagnetic (Ni) thin films, where it was suggested that femtosecond laser-induced ultrafast demagnetization process was responsible for the THz pulse emission (Beaurepaire et al., 1996). From the subsequent studies (Hilton et al., 2004), it was realized that only weak THz radiation is possible from such a process. The role of the ultrafast demagnetization process for THz generation from single-layer FM films was further investigated with respect to the thickness of the film to find an optimal efficiency for a specific thickness (Kumar et al., 2015). Zhang and coworkers attributed anomalous Hall effect for the generation of THz radiation from thin single ferromagnetic, (Fe_x_Mn_1−x_)_y_Pt_1−y_ layers (Zhang et al., 2019). Similarly, a few other studies in the literature have indicated the generation of a very weak THz radiation from femtosecond laser-excited NM metal-based structures (Kadlec et al., 2004). The issue of low efficiency was quickly resolved as soon as FM/NM-based metallic heterostructures were experimented upon using the femtosecond laser excitation (Kampfrath et al., 2013a), where the ISHE was considered to be the underlying mechanism (Saitoh et al., 2006). Specifically, the ultrafast laser pulse-induced nonequilibrium quenching of magnetization occurs in the ferromagnetic layer. Consequently, the formation of superdiffusive spin current takes place because of the higher mobility and lifetime of hot majority spins (Battiato et al, 2010, 2012; Melnikov et al., 2011). In the NM layer, strong spin-orbit coupling converts the spin current into transient charge current, which emits THz radiation. Not only a high generation efficiency but also broad bandwidth of the generated THz radiation became realizable (Seifert et al., 2016). Here, the spin Hall angle in the NM heavy metal layer, the spin polarization in the FM layer, the individual film quality, and thicknesses in the heterostructure are the major contributing material parameters. In principle, the bandwidth of the THz radiation generated from these sources is limited by the duration of the excitation pulse only (Seifert et al., 2016).

THz generation from the above mentioned spintronic heterostructures shows a nonsaturating behavior, i.e., nearly linearly increasing THz power with respect to the excitation power (Torosyan et al., 2018; Seifert et al., 2016, 2017a, 2018; Wu et al., 2017). By using proper thicknesses and combinations of the FM/NM layers in their heterostructures and the optical conditions in the experiments, THz electric field strengths of a few hundreds of kV/cm along with broad bandwidth are achievable (Seifert et al., 2017a). Conventionally, high power THz radiation is generated from amplified laser pulse-excited nonlinear crystals such as lithium niobate (Fülöp et al., 2010), organic materials (Hauri et al., 2011), and the dual-color air plasma (Bartel et al., 2005; Kress et al., 2004; Kumar et al., 2021b, 2021c). For the realization of high-power THz radiation from air plasma, it requires very high-energy pump pulses. Nonlinear optical materials like lithium niobate crystal and organic crystals, which can sustain hard pumping and provide intense THz pulses, have limitations in terms of strict phase-matching conditions, absorption at THz frequencies, etc.(Fülöp et al., 2020). The THz radiation with its electric field strength as high as a few MV/cm is also possible these days (Oh et al., 2014). In principle, the efficiency of any solid-state material-based THz source is ultimately limited by the associated optical damage threshold.

The spintronic THz sources, very often, have been tested with low pulse energy femtosecond lasers for achieving broad bandwidth (Torosyan et al., 2018; Seifert et al., 2016). In a very few cases, they have also been tested with the amplified laser pulses (Seifert et al., 2017a), for achieving high power THz radiation. Beyond a certain value of the excitation power, spin accumulation in the NM layer contributes to a weak saturating behavior (Yang et al., 2016; Kampfrath et al., 2013a). Furthermore, like the nonlinear crystals, spintronic THz sources can be used only before their optical damage threshold. As compared to others, spintronic-based THz emitters have a large scope in terms of the choice of the materials that can be used, better tunability in polarization, bandwidth, and power. Optimization through excitation wavelength, sample geometry, applied magnetic field, etc. can be achieved easily (Torosyan et al., 2018; Seifert et al., 2016, 2017a, 2018; Wu et al., 2017; Herapath et al., 2019; Kong et al., 2019; Chen et al., 2019; Feng et al., 2018; Kumar et al., 2021a). In the current study, we have experimentally determined the optical damage threshold of many popular spintronic THz emitters on different substrates by using femtosecond amplified laser pulses at 800 nm, which has not been reported explicitly in the literature, hitherto. Fe and CoFeB have been used for the FM layer, and Pt and Ta (in α-phase) have been used as the NM layer in the bilayer spintronic heterostructures. These heterostructures have been tested for optimal THz power generation by using various combinations of the FM and NM layer thicknesses. Ultrafast demagnetization and inverse spin Hall effect have been invoked for the THz generation mechanism. Ta in its specific α-phase has been used in our heterostructures, in which its role as a buffer layer and an efficient ISHE material has been determined. For Fe/Ta bilayer heterostructure on quartz substrates, the optical damage threshold is the highest at ∼85 GW/cm^2^, which is quite comparable with that of the popularly used lithium niobate crystal. The THz generation efficiency of the thickness optimized Fe/Pt bilayer structure is found to be even better than the dual-color air plasma-based THz source for excitation pump energy levels below ∼0.5 mJ. While the CoFeB/Pt is a more efficient source than Fe/Pt, being consistent with the literature, we find it to be opposite in the case with Ta as the NM layer, i.e., THz power generation efficiency of Fe/Ta is much higher than that of the CoFeB/Ta. Differences found in the optical damage threshold of a particular heterostructure on different substrates are consistent with the differences in their THz generation efficiency. The reason has primarily to do with the optical power absorbed by them, a weak absorber has lower THz generation efficiency at any given excitation power, and also, it has a higher damage threshold.

## Results and discussion

**Figure 1:**
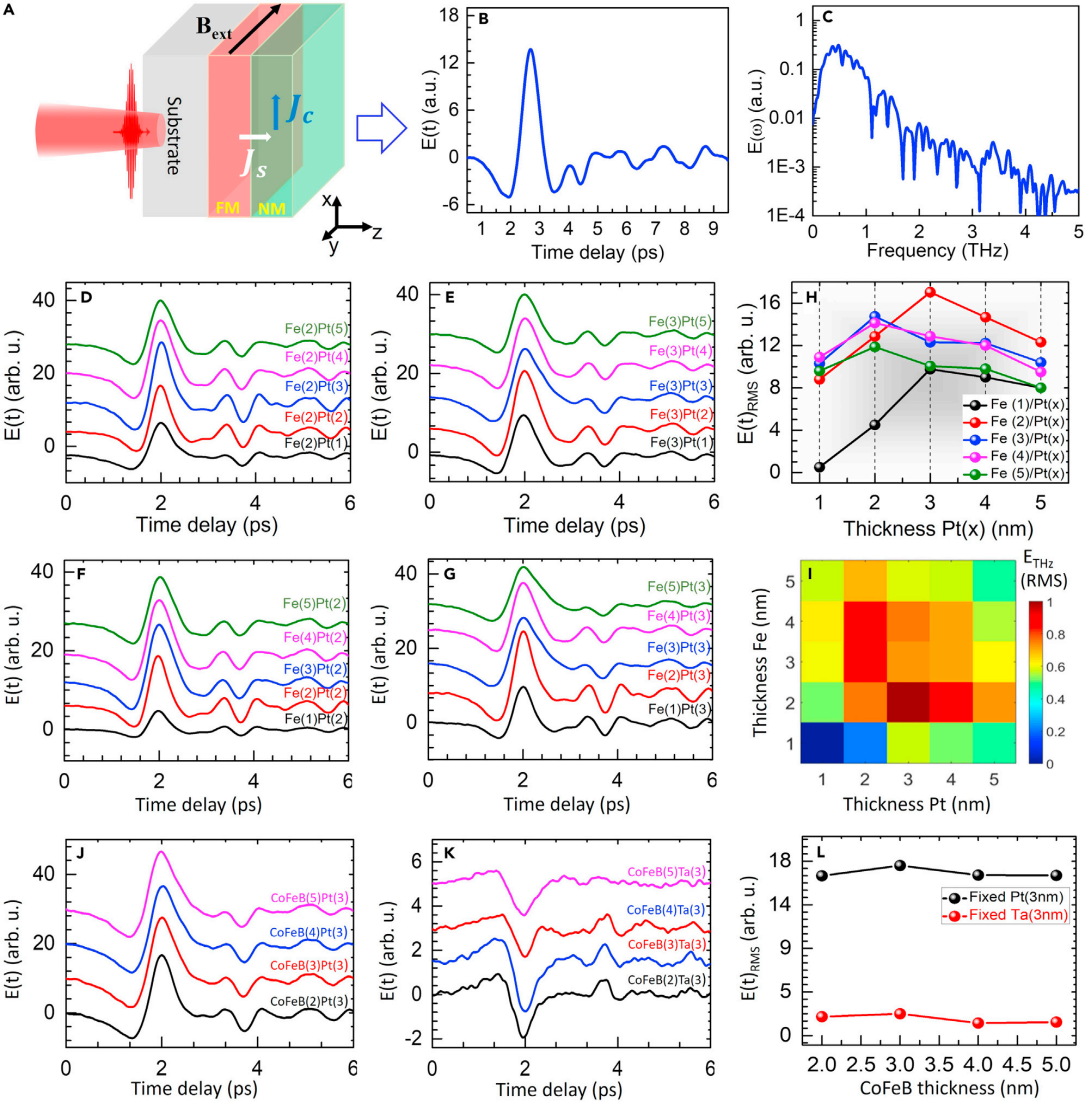
THz pulse generation from femtosecond NIR (800 nm) pulse irradiated FM/NM bilayer-type spintronic emitters deposited on quartz substrates at the excitation pulse energy (fluence) of ∼0.35 mJ (1.2 mJ/cm^2^) (A) Schematic of the optical pulse excitation and THz pulse generation. B_ext_ is the external magnetic field, J_s_ is spin current, and J_c_ is charge current density. (B) Typical time domain THz signal, E(t) detected by electro-optic sampling. (C) The Fourier transformed spectrum, E(ω). (D–I) THz signals from Fe/Pt heterostructures for various combinations of thicknesses (nm) of the Fe and Pt layers. (J–L) THz signals from CoFeB/Pt and CoFeB/Ta bilayers for various thickness combinations of the two layers.

**Figure 2:**
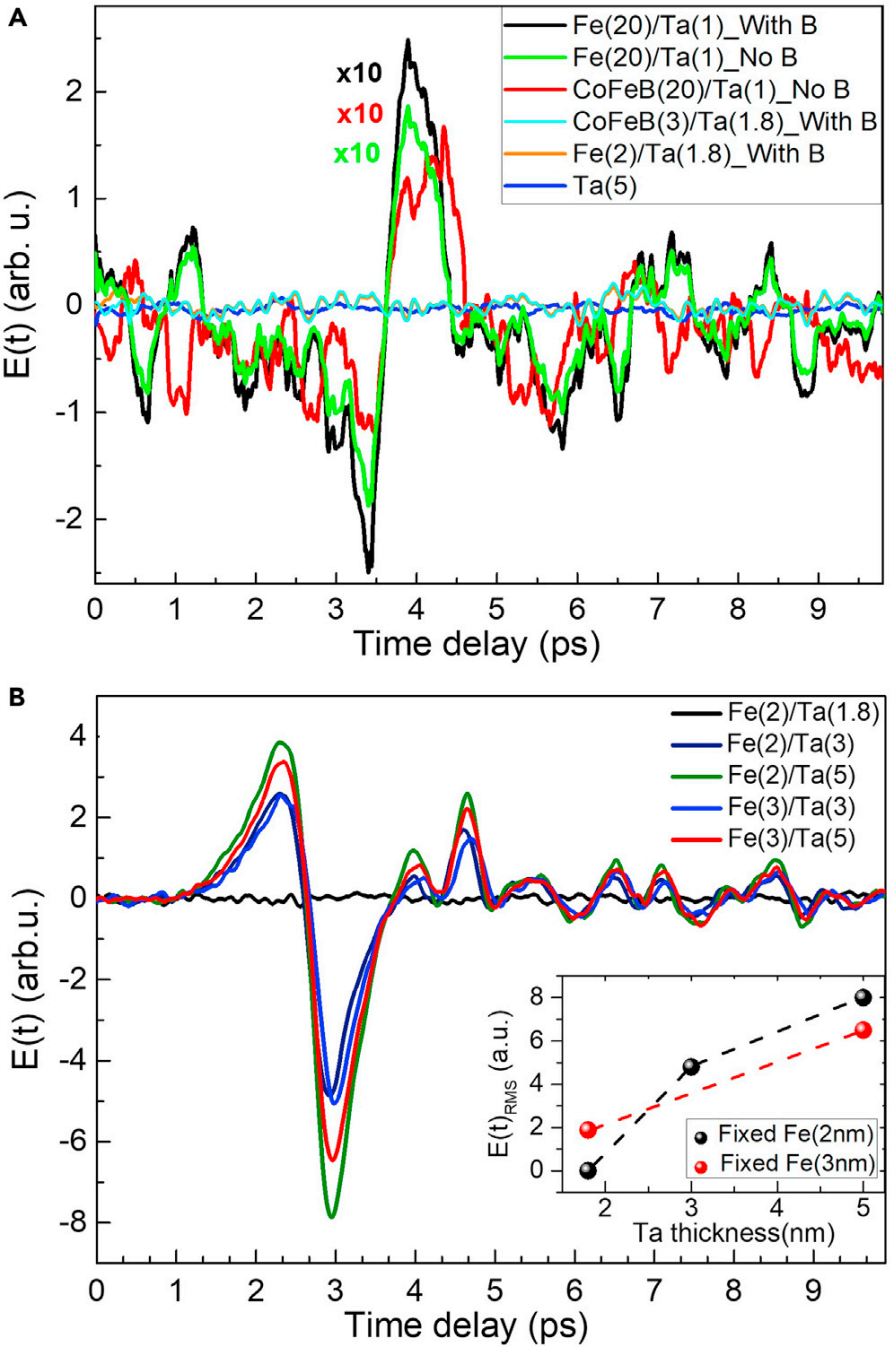
THz generation from FM/NM bilayer heterostructures and their individual constituents (A) THz signal generated from individual FM and NM layers. A very thin Ta layer (thickness ∼1.5 nm) was used as the capping layer for the FM film on quartz substrate. (B) The THz signal from Fe/Ta bilayers to obtain the optimized thickness of the Fe and Ta layers. Inset: The RMS amplitude of the THz field varying with the increasing thickness of the Ta layer at fixed thicknesses of the Fe layer (2 nm and 3 nm).

**Figure 3:**
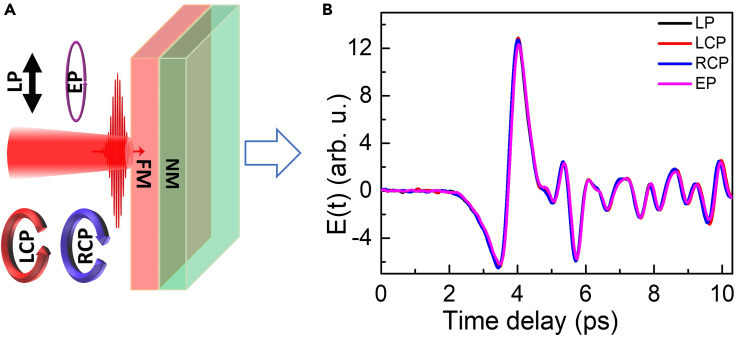
Excitation pulse helicity-dependent THz emission from Fe(2)/Pt(3) bilayer heterostructure (A) Schematic of the experiment. (B) Excitation polarization-dependent THz time domain signals. LP: linear polarization, LCP: left-handed circular polarization, RCP: right-handed circular polarization, EP: elliptical polarization.

**Figure 4:**
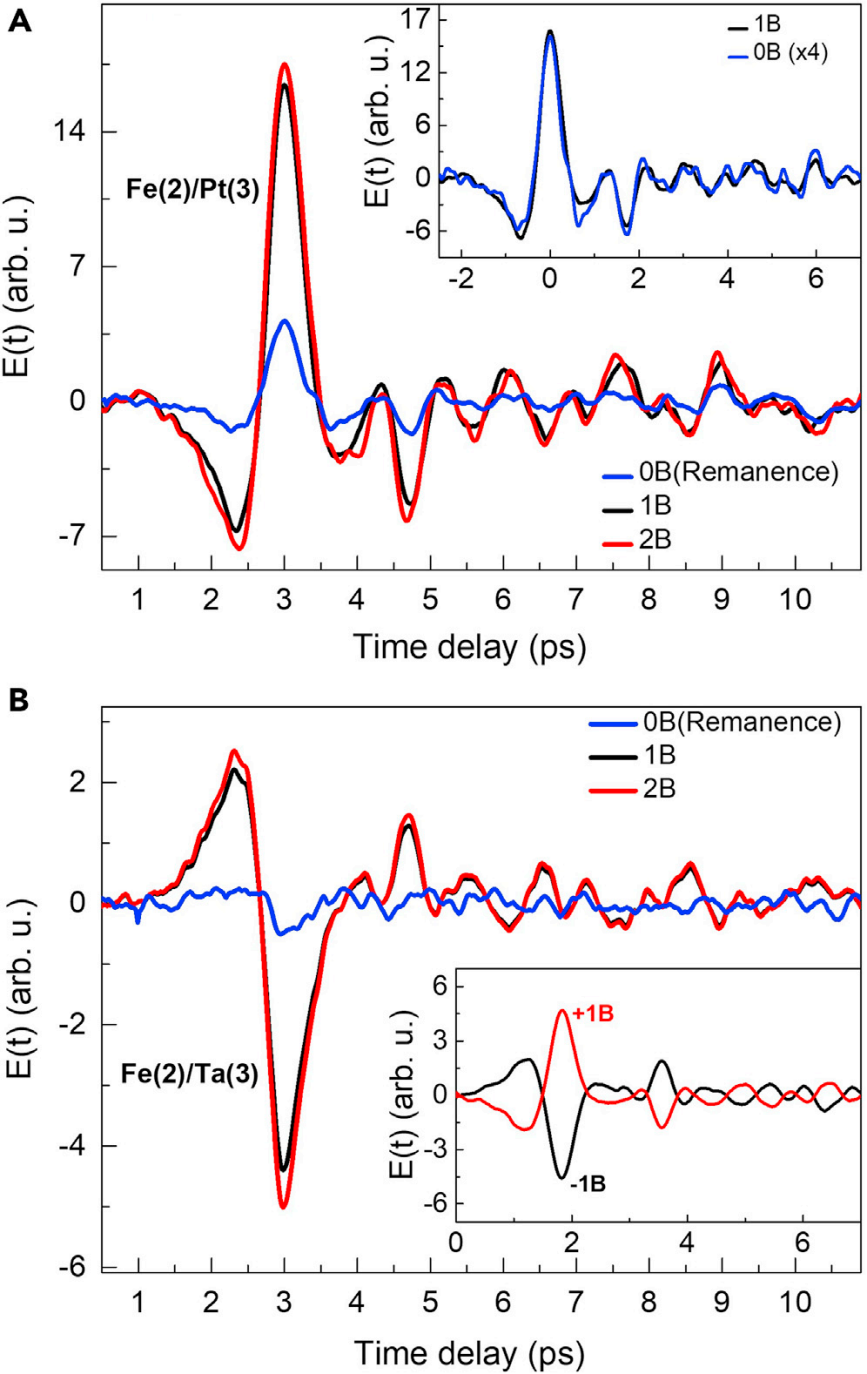
THz generation from FM/NM spintronic emitters in the presence of external magnetic field varying from 0B to 2B (A) Time domain THz traces from Fe(2)/Pt(3). Inset: Comparison between applied and no applied magnetic fields. The signal for 0B has been magnified by 4 times. (B) Time domain THz traces from Fe(2)/Ta(3). Inset: polarity reversal of the THz signal by reversing the magnetic field direction as per the ISHE in Equation (1).

**Figure 5:**
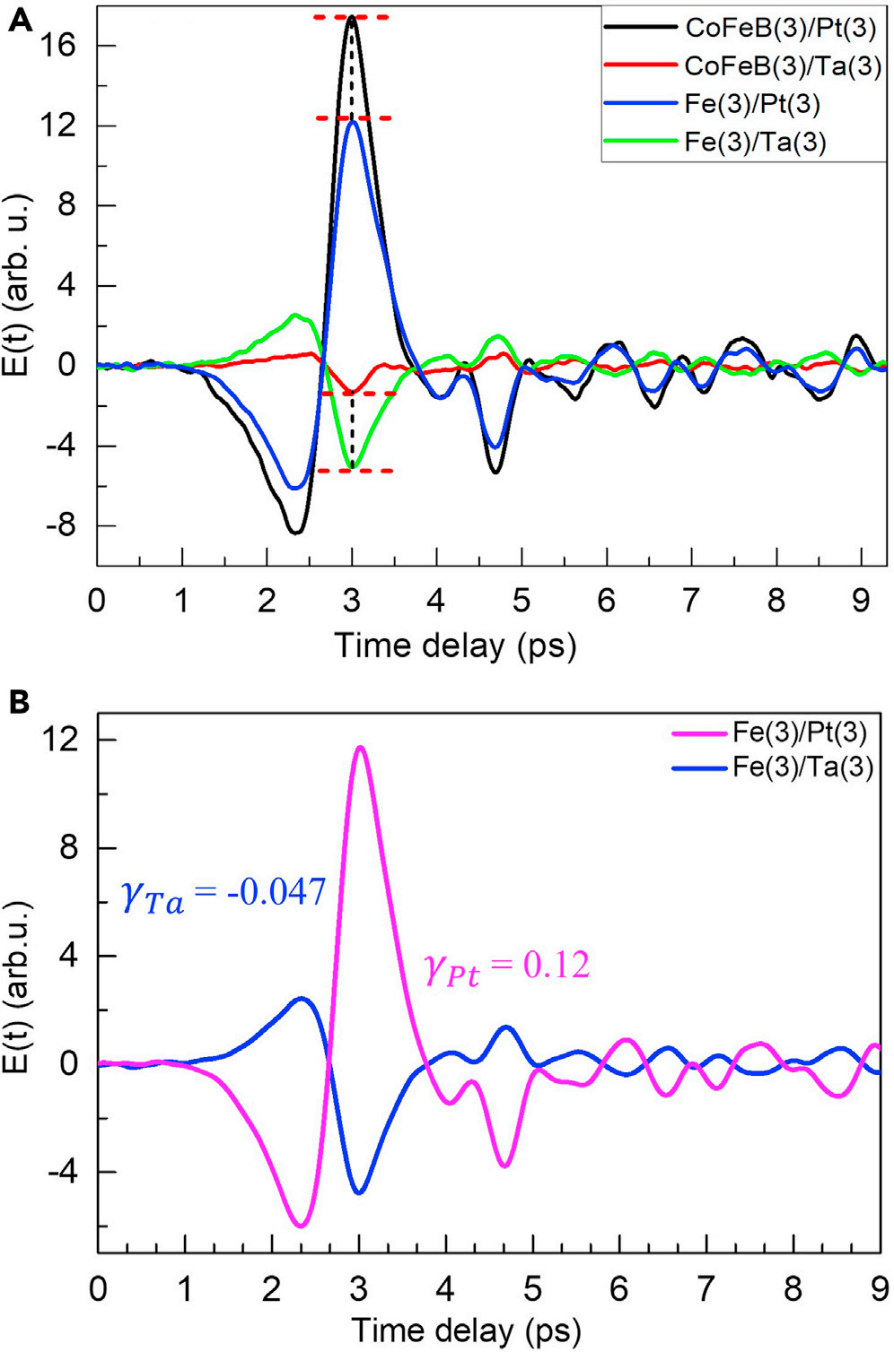
THz signal comparison and estimated spin Hall angle values from the THz signal comparison of FM/NM spintronic emitters (A) Comparison of the magnitude of the THz signals from Fe/Pt and Fe/Ta with that from the CoFeB/Pt and CoFeB/Ta bilayer heterostructures. (B) Estimation of spin-Hall angle γ in α-phase Ta of the Fe(3)/Ta(3) bilayer heterostructure from the comparison between the THz signals from Fe/Pt and Fe/Ta.

All the results discussed through Figures 1, 2, 3, 4, and 5 have been obtained at a fixed excitation pulse energy (average power) of ∼0.35 mJ (350 mW) or the fluence of ∼1.2 mJ/cm^2^. Figure 1 summarizes the outcomes on the THz generation efficiency from various thickness combinations in the bilayer FM/NM heterostructures on quartz substrates that are fabricated by magnetron sputtering in ultrahigh vacuum (see the Method details). FM layer is of either Fe or CoFeB, and the NM layer is of either Pt or Ta, thereby allowing four possible FM/NM combinations for experimentation on them. The Ta layer is grown in its α-phase (see Figure S1). These results in Figure 1 provide the optimized thicknesses and their combinations for the total thickness of the bilayer structure such that maximum THz power is produced from them under the same conditions with the optical excitation, room temperature and humidity, optical alignment, and the detection by electro-optic sampling. A schematic of the THz pulse generation following excitation by a femtosecond laser pulse at near-infrared (NIR) is shown in Figure 1A. A representative THz time-domain signal, E(t), and its Fourier spectrum, E(ω), are presented in Figures 1B and 1C, respectively, generated from Fe(3)/Pt(2) bilayer heterostructure. All the THz generation and detection measurements reported in this paper have been done under room temperature and humidity (∼50%) conditions that are realistic for all practical purposes, mainly the applications in stand-off detection and screening. Hence, the strong far IR absorption lines due to the characteristic water absorption bands (Xin et al., 2006) in the THz spectrum can be seen from Figure 1C. The limited THz bandwidth (∼0.2–5 THz) is mainly due to the ZnTe crystal used in our electro-optic sampling setup for THz pulse detection. The magnitude of the THz time domain signal can be improved immensely if the enclosure of the experimental setup is purged with dry air. The enhancement factor may vary depending on the available bandwidth of the generated THz radiation. However, for consistency, all the results below have been obtained without any purging.

The femtosecond pump excitation (z-direction) of the FM layer, which is kept magnetized by B_ext_ along -y-direction, produces a spin current. The spin current J_s_ in the z-direction gets injected into the NM layer of the heterostructure. Due to strong spin-orbit coupling in the NM layer by virtue of the ISHE (Saitoh et al., 2006), a charge current J_c_ is produced in the x-direction as shown in Figure 1A. Note that the magnetization direction is defined by the external magnetic field, B_ext_, which is kept just above the saturation magnetization field of the FM layer. Briefly, the THz pulse generation process through ISHE in the FM/NM bilayer spintronic heterostructure is described here. NIR femtosecond excitation pulse stimulates the electrons in the FM metal layer from states below the Fermi level to above it, creating a non-equilibrium hot electron distribution (Battiato et al., 2010). The FM metal is such that there is a difference in its spin-up and spin-down electron densities and their transport properties in the respective bands (Knorren et al., 2000). This difference helps in producing a spin-polarized current (J_s_) to be injected into the NM layer employing a superdiffusive process if an appropriate thickness of the FM layer is used (Battiato et al., 2010; Melnikov et al., 2011). Since the NM layer of the spintronics heterostructure is a heavy metal with high intrinsic spin-orbit coupling, opposite polarity spins are deflected in opposite directions by an amount proportional to the spin-Hall angle (γ) and hence produce a charge current density (Hoffmann, 2013), given by the following relation (Kampfrath et al., 2013a; Wu et al., 2017).

(Equation 1) $$\;\vec{J_{c}}=\;\gamma .\;\left(\;\vec{J_{s}}\times \hat{m}\right)$$

Here, $\hat{m}=\vec{M}/\left|\vec{M}\right|$ is the magnetization direction. Therefore, the in-plane transient charge current J_c_ in the NM layer generates a pulse of THz radiation which has been measured by electro-optic sampling in our experiments.

Figures 1D–1I show the results obtained from Fe/Pt structures on quartz substrates for different thicknesses of the two individual layers in nanometers (nm) as mentioned inside small parentheses. The raw THz signals obtained for fixed Fe thickness and varying Pt thickness are shown in Figures 1D and 1E. Similarly, raw results for fixed Pt layer thickness and varying Fe layer thickness are presented in Figures 1F and 1G. For clarity, the consecutive time domain traces in Figures 1D–1G have been shifted vertically by a constant amount. As can be seen from Figures 1H and 1I that the root mean squared (RMS) amplitude of the THz field (E(t)_RMS_) is dependent on the thickness of the individual layers, there is an optimum value of the thickness where maximum THz power is generated. It is clear from the thickness-thickness color intensity plot in Figure 1I that the optimum thickness of the Fe layer is ∼2 nm and that of the Pt layer is ∼3 nm, and for other values of thicknesses of the two layers, the maximum THz generation is found to be for thickness ∼5 nm of the combined structure. These results are consistent with the literature (Torosyan et al., 2018). Therefore, for referral purposes later on in the manuscript, the optimized Fe/Pt heterostructure on quartz contains a 2-nm-thick Fe layer and a 3-nm-thick Pt layer. The optimum thickness of the Fe layer is very close to its critical thickness (∼1.5 nm) for the change in the direction of the magnetic easy axis in the layer (please see the Figure S1). Determination of the thickness-dependent performance for THz emission was necessary because the optical damage threshold measurements are conducted on the thickness-optimized heterostructures.

Various parameters such as spin current generation in the FM layer, spin-to-charge current conversion and spin accumulation in the NM layer, optical and THz absorption in the total thickness, etc. contribute to the overall THz generation efficiency of the bilayer FM/NM heterostructures (Papaioannou and Beigang, 2021). From Figure 1H, it can be seen that for a fixed thickness of the Fe layer, there is an optimum thickness of the Pt layer at which maximum THz generation can be obtained. Efficient conversion of the spin-polarized current into the transient charge current in the NM layer is necessary for maximum THz generation efficiency (Haney et al., 2013). The decrease in the THz signal at lower thicknesses is due to the limited spin decay length in the NM material. The optimum thickness of the Pt layer for generation of the highest THz signal from the heterostructure is about 3 nm, which is just above the spin decay length of ∼1 nm in Pt (Torosyan et al., 2018; Seifert et al., 2016, 2017b). A dramatic change in the THz RMS amplitude takes place with the thickness of the Fe layer changing from 2 nm to 1 nm in Figure 1H. This is attributed to the change in magnetization easy axis going from being in-plane to out of plane, below a certain thickness (see the Figure S1). From Figures 1H and 1I, it is clear that the optimum thickness of the Fe layer is about 2 nm. Similarly, it can be seen from the figure that, at smaller thicknesses of the Pt layer, the THz power continues to grow with the increasing thickness up till its optimum value at ∼3 nm. At the optimum thickness of the Fe layer, the decrease in the THz generation efficiency with the increasing Pt layer thickness (beyond 3 nm) can be understood in terms of a compromise between the spin-to-charge current conversion for the fixed excitation fluence and the overall THz attenuation in the bilayer heterostructure (Seifert et al., 2016). The former is due to the spread of absorbed excitation power over a larger thickness in thick films. The overall thickness beyond which the THz generation efficiency starts to decrease is ∼5 nm.

The raw THz results for fixed thicknesses of Pt or Ta layer at 3 nm while the varying thickness of FM CoFeB layer from 2 to 5 nm in the CoFeB/Pt and CoFeB/Ta bilayers are presented in Figures 1J and 1K, respectively. The RMS values of the THz signal for each sample are presented in Figure 1L. It can be seen that the optimum value of the CoFeB layer thickness is close to 3 nm beyond which there is not much improvement in the THz generation efficiency, and also, the THz attenuation in the CoFeB does not increase much with its thickness up to 5 nm. In these cases, we have already used the optimum thickness of the Pt layer of ∼3 nm. Our results (Figure 2) indicate that the optimum thickness of the Ta layer in the heterostructures is more than 5 nm, while almost no THz signal is measured from the bilayers containing Ta layer thickness below 2 nm. In addition to the opposite polarity THz signal, we also find much lower THz generation efficiency of the CoFeB/Ta than the CoFeB/Pt bilayers (Figures 1J and 1K). Both of these effects can be attributed to the opposite sign and much smaller spin-Hall angle, respectively, in the α-phase Ta as compared to Pt (Kumar et al., 2018; Sinova et al., 2015).

The ultrafast demagnetization is also a process, which under femtosecond excitation of FM layers generates THz radiation and has been reported in several studies (Beaurepaire et al., 2004; Kumar et al., 2015; Huang et al., 2020). From our results presented in Figure 2A, we intend to disseminate a conclusion that the FM and NM layers individually contribute very weakly to the THz generation process. As discussed later, in comparison, the THz signal produced by ISHE is about two orders of magnitude stronger from the bilayer heterostructures. From Figure 2A, we can see that no THz signal is produced at all from Ta(5), Fe(2)/Ta(1.8), and CoFeB(3)/Ta(1.8) samples. Clearly, the THz generation is not substantially enough either through ultrafast demagnetization in thin FM layers or through ISHE in FM/NM bilayers without having an appropriately thick NM layer. A weak THz generation from thick FM layers through ultrafast demagnetization is evident from our results also as presented in Figure 2A for the Fe(20)/Ta(1) and CoFeB(20)/Ta(1) samples, where the signals have been magnified by a factor of 10 for better visualization. These results are consistent with the literature (Huang et al., 2020). Here, in all of these cases, a very thin Ta layer has been utilized as a capping layer (thickness of ∼1 nm) for the FM film. For such a thin Ta layer, it does not contribute as the NM layer in the otherwise FM/NM heterostructures of Figure 2B also having no THz signal from Fe(2)/Ta(1.8). Furthermore, almost no role of the external magnetic field can be noted in Figure 2A for the weak THz signal generation from FM films (Huang et al., 2019, 2020).

THz generation by ISHE in thin-film Fe/Ta heterostructures can be observed only if the Ta layer thickness is larger than 2 nm. The corresponding results have been presented in Figure 2B, where data are shown for Fe layer thicknesses 2 nm and 3 nm, while Ta layer thickness varying from 1.8 nm to 5 nm. These results again confirm that the optimized thickness of the Fe layer for maximum THz signal is ∼2 nm. As shown in the inset of Figure 2B, the THz signal continues to grow with the increasing Ta layer thickness. While comparing the results for the Fe/Pt (Figure 1) and Fe/Ta (Figure 2B), we find that the optimum thickness of the α-phase Ta layer is much larger than the Pt layer. It is noteworthy to note from the results discussed above that the demarcation line for the thickness of the α-phase Ta layer below which it can be used as a capping or buffer layer is ∼2 nm. However, it may vary depending on the different phases of the Ta (Kumar et al., 2018). Besides the fact that the spin Hall angle in Ta (all phases) is opposite in sign to that in the conventionally used Pt layer, depending on the phase, its spin Hall angle and resistivity both span over a large range (Kim et al., 2015; Bansal et al., 2017; Kumar et al., 2018; Niimi and Otani, 2015). The THz generation efficiency through the ISHE is directly proportional to the spin Hall angle, spin-mixing conductance, and spin diffusion length, while it is inversely proportional to the resistivity of the material layer (Dang et al., 2020; Seifert et al., 2016). All these parameters need to be optimized so as to constitute a correct figure of merit of the most efficient spintronic THz emitter. Ta in β-phase has a higher value of spin Hall angle (∼−0.15) (ref. (Liu et al., 2012)) than Pt (∼0.12) (ref. (Obstbaum et al., 2014)). However, due to high resistivity in this phase, it is not a good substitute for the highly expensive Pt layer. Owing to the reduced charge current generation following optical pumping, the conversion efficiency into THz radiation is highly limited in the high resistive β-phase of Ta (Seifert et al., 2016).

The presence of an interface and intermixing at the interface can contribute to the generation of THz signal through various mechanisms, which can be probed through excitation pulse helicity-dependent measurements. For example, the THz generation process based on ISHE does not depend on helicity, whereas the spin-dependent photogalvanic effect (SDPE) is dependent on the helicity of the excitation pulse (Li et al., 2019). Our experimental results presented in Figure 3 suggest that only ISHE is responsible for the THz generation from FM/NM heterostructures. Here in Figure 3, the results have been presented for optimized Fe(2)/Pt(3) bilayer for linearly polarized (LP), elliptically polarized (EP), left circularly polarized (LCP), and right circularly polarized (RCP) excitation light. Therefore, the contribution from the SDPE into the THz pulse generation from FM/NM bilayers is negligibly small. We may also point out that a recent report on the THz pulse generation from optically excited antiferromagnetic/nonmagnetic film heterostructure arises due to a different mechanism, where helicity-dependent outcomes can be possible (Qiu et al., 2020).

As per Equation (1) for the ISHE being responsible for the THz pulse generation, the magnetization of the FM layer can be used as a control to manipulate the amplitude and polarization of the emitted THz radiation (Qiu et al., 2018; Torosyan et al., 2018). In Figures 4A and 4B, we have shown the time domain THz electric field signals from Fe(2)/Pt(3) and Fe(2)/Ta(3) samples, respectively, recorded with and without the external field, B_ext_. For the Fe layer, the saturation magnetic field (coercive field) is ∼50 Oe as confirmed from the in-plane M–H measurements (see Figure S1). Therefore, for the magnetic field effect, we have shown the results for three values of B_ext_, i.e., 0B (no field), 1B ∼200 Oe, and 2B ∼400 Oe. In the first case (0B), only the effect of any remanence field is observed in terms of a small magnitude THz signal (Kampfrath et al., 2013a), due to the pinned magnetic domains that could avoid reorientation after removal of the external field. For the other two field values (1B and 2B), the THz signal is already saturated. Except for the change in the magnitude, the temporal profiles of the THz pulses are exactly the same irrespective of the external magnetic field value (see inset of Figure 4A). As shown in the inset of Figure 4B, the polarity of the generated THz signal can be reversed by flipping the direction of the external magnetic field as per the ISHE in Equation (1) (Seifert et al., 2016).

Now in Figure 5, we compare the THz generation efficiency of the near thickness-optimized Fe/Pt, Fe/Ta, CoFeB/Pt, and CoFeB/Ta bilayer heterostructures having the same thicknesses of the FM and NM layers in each of the combinations and under the same experimental conditions. The CoFeB(3)/Pt(3) heterostructure provides ∼40% stronger THz emission than that from Fe(3)/Pt(3). The enhanced THz signal from the prior can be attributed to the larger spin injection into the NM layer and is consistent with the literature (Seifert et al., 2016). Interestingly, the case is entirely reversed for the Fe/Ta and CoFeB/Ta bilayers, which contain Ta as the NM layer. Another highlight from Figure 5A is that for the same α-phase Ta as the NM layer in the heterostructures, the THz signal from Fe(3)/Ta(3) is stronger by ∼300% than that in CoFeB(3)/Ta(3). This observation indicates that the spin injection efficiency in the CoFeB(3)/Ta(3) might have got severely affected by the seemingly larger crystalline mismatch and roughness at the FM/NM interface, both of which essentially affect the spin mixing conductance $\left(g_{↑↓}\right)$ and the spin-Hall angle at the interface (Dang et al., 2020).

The spin mixing conductance $g_{↑↓}$ is a measure of the spin current injected from the FM layer into the NM layer. Assuming a simple precession motion, the spin current density is related to the spin mixing conductance through a relation given by (Kumar et al., 2018; Mosendz et al., 2010): $J_{s}=\left(1/2\pi \right)e\omega \text{Re}\left(g_{↑↓}\right)\text{sin}\left(\theta_{c}\right)$, where, $e,\;\omega ,\;\text{Re}\left(g_{↑↓}\right),\;\text{and }\theta_{c}$ are the electronic charge, the angular frequency of spin dynamics, the real part of spin mixing conductance, and the magnetization precession angle, respectively. Therefore, a higher value of $\text{Re}\left(g_{↑↓}\right)$ ensures an enhanced THz emission through Equation (1). The values of the spin mixing conductance reported in the literature are ∼3.5–6×10^19^ m^−2^ for CoFeB/Pt (Zhu et al., 2019; Conca et al., 2017; Kim et al., 2014), ∼3–5×10^19^ m^−2^ for Fe/Pt (Conca et al., 2016; Papaioannou et al., 2013), and ∼0.7–1.4×10^19^ m^−2^ for CoFeB/Ta (Conca et al., 2017; Kim et al., 2014). For the CoFeB/Ta case in references (Conca et al., 2017; Kim et al., 2014), Ta in its β-phase was used (Conca et al., 2017; Kim et al., 2014). Similar values for Fe/Ta could not be found in the literature. Moreover, the FM/NM spintronic heterostructures containing α-phase Ta have not been studied much in the literature. The α-phase of Ta is more metallic than the β-phase and hence can be better suited for the THz applications though it has comparatively a slightly smaller spin-Hall coefficient (Kumar et al., 2018). The experimentally observed small relative change in the THz signal from CoFeB/Pt as compared to Fe/Pt (Figure 5A) seems consistent with the corresponding values of the $g_{↑↓}$. However, the much larger THz signal from Fe/Ta than the CoFeB/Ta demands that the corresponding $g_{↑↓}$ value in Fe/Ta should be higher than that of the CoFeB/Ta. Our THz experiments suggest a higher value of $g_{↑↓}$ for the Fe/Ta as compared to CoFeB/Ta. The conclusion from the results in this part is that Pt should be favored with CoFeB, while α-phase Ta should be favored with Fe in their FM/NM bilayer heterostructures for best THz generation performance.

Besides the electronic mechanisms (Saitoh et al., 2006), a comparison in the magnitude of the THz signals from two FM/NM combinations can be used to quantify the spin-Hall angle in a given NM layer (Seifert et al., 2018). For example, the THz generation efficiencies of CoFeB/Pt and CoFeB/W were compared to determine the spin-Hall angle in W to be γ ∼ −0.056, which was very close to its exact value (Mondal et al., 2017). We have used the known value of γ = 0.12 in Pt (Seki et al., 2008; Sinova et al., 2015; Obstbaum et al., 2014) to estimate the same in α-phase Ta by comparing the THz signal strengths from Fe/Pt and Fe/Ta in Figure 5B, where the same thickness FM layer is used. From the ratio between the RMS values of the corresponding THz signal strengths in Figure 5B, γ_Ta_ ∼ −0.047 has been estimated. Of course, for the exact determination of the γ value in α-phase Ta, one needs information about the corresponding $g_{↑↓}$ value and other material parameters, precisely (Seifert et al., 2018). Nevertheless, our estimated value of γ_Ta_ ∼ −0.047 here closely matches with the value from the literature (Kumar et al., 2018), where spin-torque ferromagnetic resonance study on α-phase Ta containing NiFe/Ta structure was carried out.

**Figure 6:**
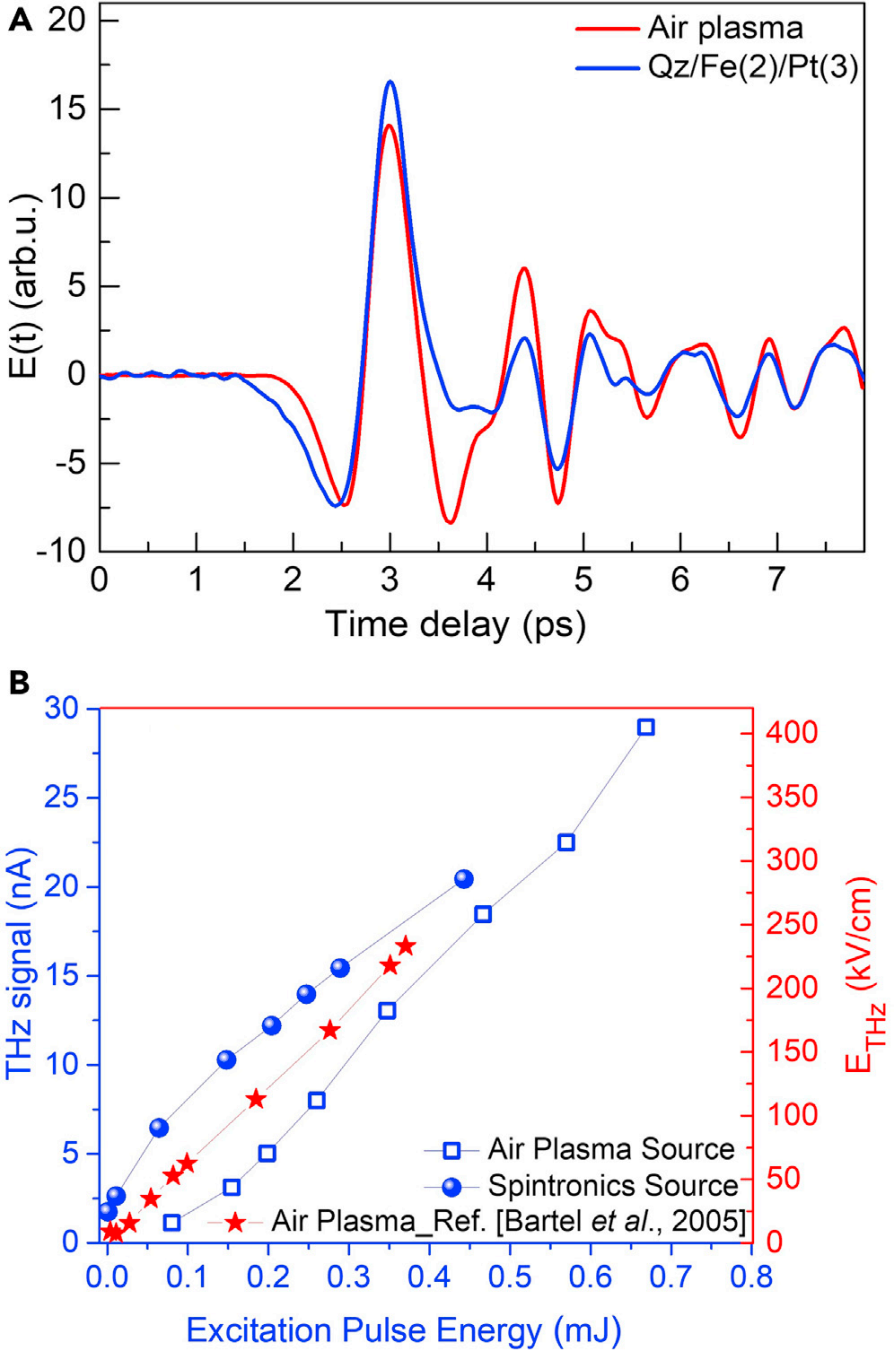
Comparison of THz emission efficiency from dual-color air plasma source to that of spintronic emitter under same excitation power (or pulse energy) and other optical conditions (A) Comparison between the magnitudes of the time domain THz traces from dual-color air plasma source and thickness-optimized Fe/Pt spintronic emitter. (B) Amplitudes of the THz signals from the air plasma source (blue squares) and the spintronic source (blue spheres) for various excitation pulse energies in our experiments. The graph for THz electric field in kV/cm (red stars) for an air plasma source is drawn from Ref. (Bartel et al., 2005), for a comparison.

For a quantitative assessment of the THz generation efficiency of the above spintronic emitters, here, we compare the THz signal strengths with a more standard high-power THz source, i.e., the dual-color air plasma source. To do so, excitation fluence-dependent measurements were carried out on both types of sources using configurations 1 and 2 of the experimental setup as shown in Figures S2 and S3, respectively. In Figure 6A, a comparison between the THz signals from the thickness optimized Fe(2)/Pt(3) spintronic heterostructure on quartz substrate and the dual-color air-plasma sources is shown. Here, an excitation pulse energy of ∼0.35 mJ was used for both types of sources. It can be seen that the THz emission from both sources is quite comparable. In fact, the THz generation efficiency of the spintronic source is slightly better than that of the air-plasma source at low excitation pulse energies as shown in Figure 6B, where, the excitation pulse energy dependence has been shown in a large range. It may be noted that the magnitude of the THz radiation from the spintronic source is underestimated from its actual value because of the fact that the optical gating beam size, the position of the nonlinear crystal for detection, and the optical alignment in the two configurations (1 and 2) are kept unchanged (see Figure S2).

In the absence of a THz power/energy meter, we could estimate the strength of the generated THz signal from our measurements in the units of THz electric field (kV/cm). To do so, we have compared in Figure 6B, the optical excitation energy-dependent THz signal strengths in nA (lock-in units) from the air plasma source (our experiments) with the values in units of kV/cm reported in the literature for the same source (Bartel et al., 2005). We point out that these experiments have been performed under very similar experimental conditions with the excitation pulse duration, the SHG crystal, focusing, etc. This comparison has been presented in Figure S8, where, a one-to-one correspondence between the two can be clearly seen. The data in the figure demonstrate a linear dependence of the THz field strength on the excitation pulse energy, while a small difference in the y axis intercept is to do with the difference in the optical arrangements and the electro-optic detection in the two cases. From these results, it can be concluded that at any excitation pulse energy, an original value of 1 nA corresponds to ∼17 kV/cm of THz electric field. This unit conversion has been used uniformly while comparing the results from different THz sources shown in the Figure 6. Furthermore, the peak amplitude of the THz electric field from the air-plasma source linearly increases with the excitation pulse energy in Figure 6B. The linear behavior is a good indication that the electro-optic effect in the ZnTe electro-optic crystal is still in the linear regime for the detection of such intense THz pulses (Gallot and Grischkowsky, 1999). This is because of the excitation energy values being much above the air plasma ionization threshold value (Augst et al., 1991; Bartel et al., 2005) of ∼10^14^ W/cm^2^. A slight difference in the two graphs in Figure 6B can be attributed to the differences in the experimental conditions due to room temperature, humidity, and the optical arrangements in the experimental setup.

**Figure 7:**
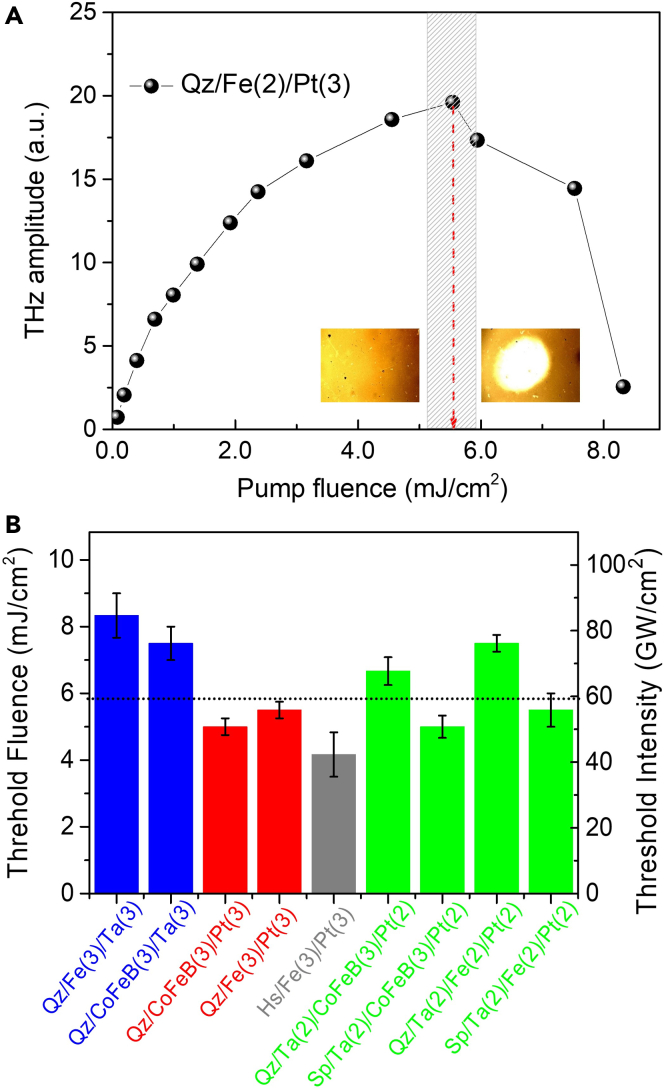
Optical damage threshold of various spintronic emitters (A) Peak THz amplitude with the increasing excitation fluence for the THz emission from a thickness-optimized FM/NM spintronic heterostructure. The dashed line in the shaded region indicates the optical damage threshold value. Insets: optical microscopy images of the sample before and after the damage. (B) Damage threshold fluence and corresponding damage threshold peak intensity of various spintronic emitters on different substrates under the normal incidence. Error bars represent the maximum possible experimental error due to uncertainty in the laser power, pulse duration, and laser beam size. The numbers inside small parentheses in the names of the sample indicate optimized thickness in nm. Qz: quartz; Sp: sapphire; Hs: highly resistive silicon.

As mentioned before, for reasonable excitation pulse energy, the spintronic source produces a slightly higher THz signal than the air plasma source (Figure 6A), and it grows nearly linearly with the excitation pulse energy. However, at very high values of the excitation pulse energy, the spintronic sources will not work because of the optical damage to the material. Here, we come to the second main point of our paper, i.e., to determine the optical damage threshold of various spintronic THz emitters, which were thickness optimized in our study. In this case of the spintronic emitters, the results and discussion are provided in terms of excitation fluence (mJ/cm^2^) or peak intensity (GW/cm^2^). The latter is simply the pulse energy/pulse duration per unit area. The excitation fluence was varied in a large range by using a neutral density filter in the excitation path while keeping the excitation beam size fixed at ∼1 mm on the sample. These measurements were done in a different configuration of the experimental setup as shown in Figure S4. In this case, the spintronic source was placed at the focal point between the two inner parabolic mirrors, and a converging optical beam through the hole of the first of those two parabolic mirrors irradiated the sample. For the Fe(2)/Pt(3) heterostructure on the quartz substrate, the THz peak amplitude with respect to the excitation fluence has been presented in Figure 7A. The magnitude of the THz signal continues to grow with the excitation fluence up to ∼5.5 mJ/cm^2^, beyond which a sudden decrease in the THz signal can be noticed. This is due to the optical damage of the spintronic material at a certain optical damage threshold value. Optical microscopy images of the sample before and after the optical damage are shown in the inset of Figure 7A. The images were taken in the transmission mode using a 10 X objective lens (please see Figure S5 for more details). The size of the damage region due to excessive heating at high-level optical excitation is close to the predetermined size of the excitation beam.

Before the optical damage starts, only a weak saturation behavior of the THz signal with the excitation fluence can be seen in Figure 7A. The irradiation time at each excitation fluence was about 5 min. The saturation can arise due to two reasons mainly, the spin accumulation and the local heating (Kampfrath et al., 2013b; Huisman et al., 2016). If the spin accumulation-induced saturation (Kampfrath et al., 2013a; Yang et al., 2016) was stronger than the latter, we should have observed saturation in the THz signal quite early in the data shown in Figure 7A. The contrasting result, therefore, suggests that the local heating-induced saturation in the THz signal is more dominant. Our observation that the THz generation from the spintronic emitters has not saturated before reaching the optical damage threshold is quite interesting. Circumventing the limitation of the optical damage will, therefore, help unlock the full potential of the spintronic THz emitters.

The optical damage threshold (ODT) of the spintronic emitters depends on the FM/NM layer combinations in the bilayer and trilayer heterostructures, their material types, and the underlying substrates. To investigate this aspect in somewhat detail, we have carried out experiments on multiple samples and on different substrates. Particularly, the ODT values have been determined for the spintronic heterostructures having optimized thicknesses of the FM and NM layers that were found out earlier in the paper. The corresponding results and the ODT values for each in mJ/cm^2^ (excitation or pump fluence) and GW/cm^2^ (peak intensity) are presented in Figure 7B. More details of the experiments are given in Figure S6. In Figure 7B, the samples have been named as per the substrates and the bi- or the tri-layer heterostructures used in them. The numbers inside small parentheses in the names of the samples represent the thickness of the individual layer in nm. The substrates used are 1-mm-thick quartz (Qz) plate, 1-mm-thick sapphire (Sp) plate, and 0.38-mm-thick highly resistive silicon wafer (Hs). The mean value of the ODT fluence for the spintronic THz emitters under the current study is about 5.8 mJ/cm^2^, and the corresponding ODT peak intensity is ∼60 GW/cm^2^. This value is only a few times smaller than the value (∼100 GW/cm^2^) for the popular lithium niobate crystal, which has been used routinely as a high-power THz source in the literature (Meng et al., 2016; Fülöp et al., 2020).

**Figure 8:**
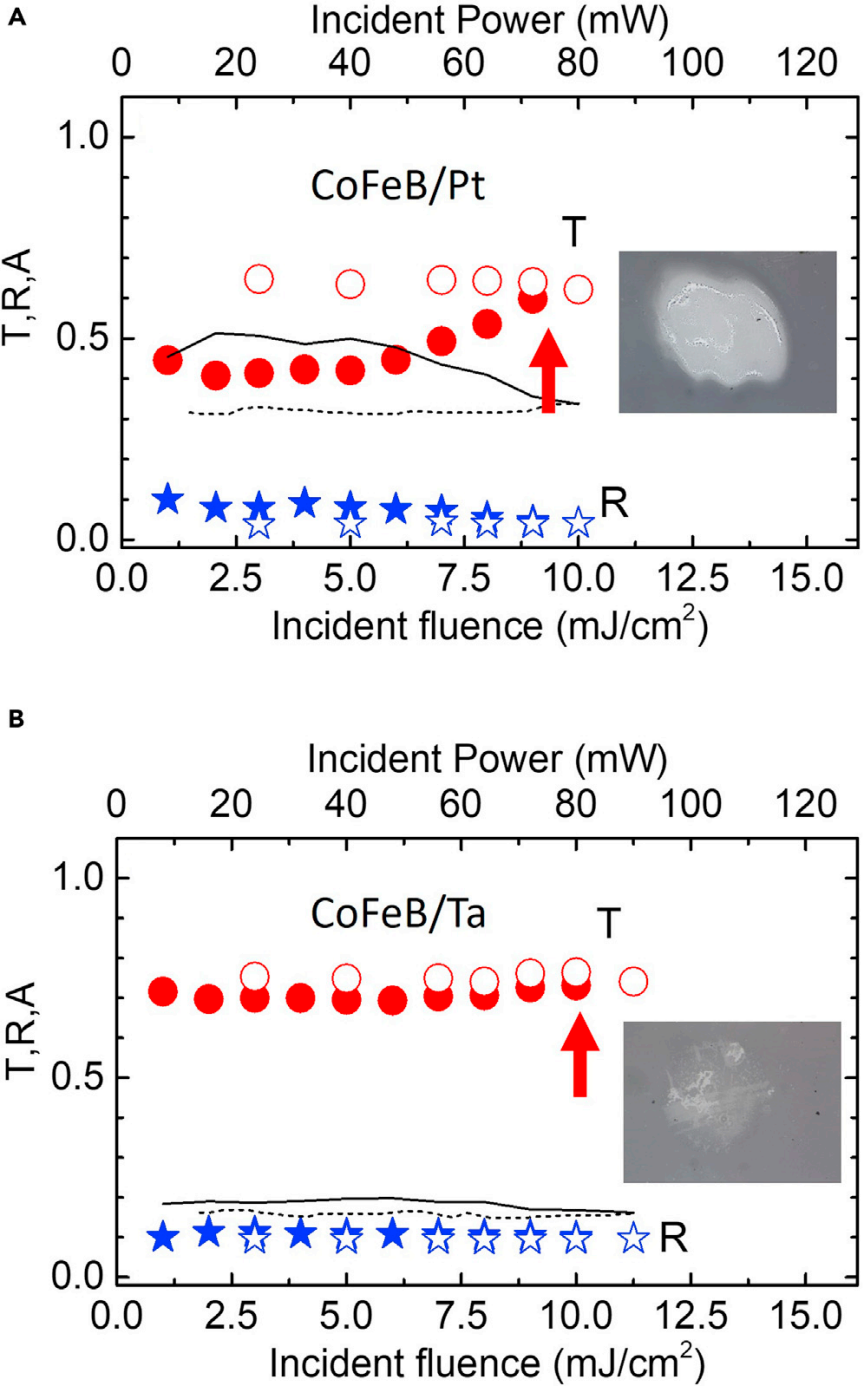
Optical transmittance (T) and reflectance (R) measurements with respect to the incident laser excitation fluence (average power). (A) For CoFeB(3)/Pt(3) sample. (B) For CoFeB(3)/Ta(3) sample. Solid symbols are for increasing fluences and open symbols are for lowering fluences. Continuous and dotted thin black curves represent the absorptance values. Insets: optical microscopy images after irradiation of the samples with excitation fluence at the optical damage threshold value indicated by the upward red arrows in the two cases.

From Figure 7B, we notice that the bilayer heterostructures with Ta as the NM layer on the quartz substrate show the highest ODT fluence value of ∼8.34 mJ/cm^2^ (peak intensity ∼85 GW/cm^2^). Therefore, quartz is suggested to be a good substrate for better heat sink management to achieve higher ODT in the spintronic THz emitters. It appears from Figure 7B that the ODT value is nearly independent of the type of the FM layer but affected by the type of the NM layer in the bilayer heterostructures on the same type of substrate. The reason for this fact is related to the overall absorptance of the sample. To substantiate this fact, we measured the fluence-dependent reflectance (R) and transmittance (T) values of the samples at various incident angles. One set of those results for the substrate-corrected R and T values at different pump fluences is presented in Figure 8 for the representative samples of CoFeB(3)/Pt(3) and CoFeB(3)/Ta(3) bilayers on the quartz substrate. The same for other two values of the angle of incidence is shown in Figure S6. In Figure 8, solid symbols are the data points taken at increasing the fluences while the open symbols are at lowering the fluences. The optical microscopy images were taken after the optical damage of the CoFeB/Pt sample at certain fluences (powers) indicated by vertically up arrows. There was a very weak damage to the CoFeB/Ta sample up to the highest experimental fluence as compared to CoFeB/Pt. Thin continuous and dotted black lines in Figures 8A and 8B are the absorptance (A = 1 - R - T) values while increasing and decreasing the excitation fluences, respectively. After the damage, while lowering the fluence values, there is no change in the R, T, and A values as can be seen from Figure 8. At any excitation fluence, the transmittance (absorptance) of CoFeB(3)/Pt(3) is smaller (higher) than that of the CoFeB(3)/Ta(3). Obviously, for the excitation wavelength of 800 nm, the lower value of absorptance of CoFeB(3)/Ta(3) than CoFeB(3)/Pt(3) is consistent with the higher damage threshold value for the prior, also can be seen through the Figure S7.

In comparison to the spintronic heterostructures on the quartz substrate, those on sapphire and HR silicon substrates show the least ODT values. This is to do with the higher optical absorption at 800 nm and less heat dissipation in those substrates. It is known that the metal-dielectric interface in spintronic THz emitters significantly improves the THz emission efficiency because of the enhanced optical absorption in such structures (Feng et al., 2018). Although a few nanometer-thick dielectric layer is almost non-dissipative for THz, it will also lead to a quicker heat deposition in the sample to cause an early optical damage of the sample. For example, as shown in Figure 7B, the optical damage threshold of the sample on SiO_2_/Si substrate is lower than that of the same on a quartz substrate. The presence of a few nm thick oxide layer hinders the transfer of heat to the more thermally conducting silicon substrate, thereby justifying the lower ODT value in this case. In our study, among the three substrates viz HR-silicon/SiO_2_, quartz, and sapphire, quartz act as the most efficient heat sink and hence provide the highest damage threshold to the thickness-optimized efficient spintronic heterostructures studied in the first part of the paper.

### Conclusions

In conclusion, optical damage threshold of various spintronic heterostructures as efficient THz emitters has been evaluated in this paper. Such a study is very important from the point of view that in recent literature on the spintronic THz emitters, they have been suggested to be the highly efficient, powerful, and broadband sources. The inverse spin-Hall effect is the main origin for high power THz generation from the bi- and tri-layer FM/NM-type spintronic THz emitters. Detailed experiments were also performed to obtain the optimized thicknesses of the individual FM and NM layers in their bilayer combinations, on which further experiments were conducted for the determination of optical damage threshold. The THz power from these sources grows nearly linearly with the excitation power before the optical damage of the material heterostructure takes place. It has been found that Fe/Ta on the quartz substrate has the highest optical damage threshold of ∼85 GW/cm^2^. From the many spintronic systems studied here, it was realized that the mean value of the optical damage threshold for such spintronic THz emitters is about 60 GW/cm^2^. These results are highly encouraging and suggest that suitable materials and their heterostructures, which provide simultaneously, high optical damage threshold and high THz generation efficiency, can be discovered.

### Limitations of the study

The FM and NM layers can be used in various possibilities to form spintronic heterostructures for efficient THz generation. In the current paper, the most popular ones have been studied. The interfacial quality of the heterostructures needs detailed investigations for enhancing the performance and determining the optical damage at various relevant excitation wavelengths.

## STAR★Methods

### Key resources table

**Table:** 

REAGENT or RESOURCE	SOURCE	IDENTIFIER
**Chemicals, peptides, and recombinant proteins**		

Iron (Fe) material sputtering Target	ACI Alloys	CAS# 7439-89-6
Platinum (Pt) material sputtering Target	ACI Alloys	CAS# 7440-06-4
Tantalum (Ta) material sputtering Target	ACI Alloys	CAS# 7440-25-7
Co_20_Fe_60_B_20_ (Cobalt Iron Boron) material sputtering Target	ACI Alloys	NA

**Software and algorithms**		

Origin 8	Origin Lab	https://www.originlab.com/

### Resource availability

#### Lead contact

Further information and requests for resources should be directed to and will be fulfilled by the lead contact, Prof. Sunil Kumar (kumarsunil@physics.iitd.ac.in).

#### Material availability

This study did not generate new unique reagents.

### Method details

#### Sample preparation and characterization

High-quality bilayer and tri-layer heterostructures of thin films containing ferromagnetic (FM) and nonmagnetic (NM) metallic materials were developed using radio frequency (RF) magnetron sputtering (AJA international). The lateral size of the films was larger than 10 mm x 10 mm and they were deposited on different substrates, 1 mm quartz, 1 mm sapphire, and 0.38 mm highly resistive silicon. The base pressure for the thin film growth was kept at 5 × 10^−8^ Torr, while the working pressure was kept in the range of 2-5 mTorr according to the optimization condition for each of the deposited material films. Prior to deposition, the substrates were cleaned by double sonication in acetone + isopropyl alcohol (IPA) to remove the contamination from their surfaces. To achieve high purity samples, we used high purity targets and pre-sputtered them to get rid of the impurities on the target surfaces before the desired film deposition. To ensure good uniformity, the substrate holder was kept rotating at a constant speed during the deposition. Different thickness combinations in the bilayer FM/NM and tri-layer NM_1_/FM/NM_2_ heterostructures were created using Fe and Co_20_Fe_60_B_20_ (CoFeB) as ferromagnetic materials and platinum (Pt) and α-phase tantalum (Ta) as nonmagnetic materials at a growth rate of 0.09 Å/sec, 0.25 Å/sec, 0.44 Å/sec, and 0.76 Å/sec, respectively. The RF power was also kept according to the optimized phase of the deposited material, i.e., 150 Watts for Ta and 100Watts for Fe, CoFeB, and Pt. The samples or the substrates were not treated with any pre- and/or post-annealing processes.

The structural and magnetic characterizations of the deposited thin film samples were obtained from X-ray diffraction (XRD), and magnetic hysteresis (M-H) measurements, respectively (see Figure S1). X-ray diffraction (XRD) and X-ray reflectivity (XRR) measurements were carried out using a PANalytical X’Pert diffractometer with a Cu-Kα source to determine the crystalline phase and purity of Pt and Ta thin films (Figures S1A and S1B). In the case of Pt, various XRD peaks have been marked with the corresponding crystallographic planes which confirm the polycrystalline phase of the film that is consistent with the literature (Pereira et al., 2013). In the XRD pattern of Ta film, a broad diffusive peak centered at angle 2θ = 38.0° is due to the Bragg reflection from (110) planes and evidently shows the growth of Ta primarily in its α-phase. The same could not be done for the ferromagnetic thin films due to the limitation with the X-ray source and rapid oxidation problem with these uncapped films (Mos et al., 2017). The crystalline α-phase of Ta is deliberately obtained by controlling the growth parameters during the deposition. The thickness and surface/interface roughness of the fabricated samples were found to be consistent with those expected from the optimized growth rate parameters in the deposition and confirmed from the XRR data fitted using the recursive theory by Parratt (1954). The surface and interfacial roughness is found to be less than ∼0.6 nm for our samples.

Since the magnetization behavior of the ferromagnetic layer in the FM/NM structures plays a crucial role in the generation of the THz radiation (Yang et al., 2016), hence, the in-plane/out-of-plane magnetic hysteresis measurements were performed using vibrating sample magnetometer (VSM) of a physical property measurement system (PPMS from Quantum Design). The results for in-plane and out-of-plane M-H measurements for Fe/Pt and in-plane for CoFeB/Pt bilayer heterostructures having fixed Pt layer thickness, but different thicknesses of the Fe and CoFeB layers are shown in Figures S1C, S1D, S1E, and S1F. It can be seen from the Figures S1C and S1D that for Fe layer thickness of 1.5 nm, it shows a non-saturating type of behavior, whereas, for higher thickness, the sample shows a clear square hysteresis with the saturation magnetic field value of <30 Oersted (Coercive field). The results for CoFeB/Pt show a saturating behavior for two values of the CoFeB layer thicknesses, while the corresponding coercive field value is ∼20 Oe. While increasing the Fe layer thickness from 1.5 nm to 3 nm, the change in the nature of the M-H curve from nonsaturating to saturating is because of the decrease in the relative contribution from the out-of-plane component of the magnetization. Clearly, for the 1.5 nm thickness, the magnetic easy-axis has a finite component in the out-of-plane, which vanishes above a critical thickness (Allenspach and Bischof, 1992). Therefore, for Fe, the critical thickness is between 1.5 nm and 3 nm, an observation that is consistent with the previous reports (Torosyan et al., 2018). Therefore, our results clearly suggest a high-quality thin films used in our experiment.

In the THz generation measurements, the applied external in-plane magnetic field was kept well above the saturation magnetization, i.e., B_ext_ > coercive field value. It may be pointed out that the out-of-plane magnetic field is not important for our THz experiments as the charge current responsible for the generation of THz radiation is insensitive to the out-of-plane magnetization direction. Therefore, for all the comparative studies performed in our paper are for FM layers having thicknesses such that the magnetic easy-axis lies in the plane and the external magnetic field parallel to it.

#### THz time-domain spectroscopy

A time-domain THz spectrometer (TDTS) was developed around a Ti:sapphire regenerative amplifier, operating at 1 kHz pulse repetition rate and providing <50 fs laser pulses centered at 800 nm. Dispersion uncompensated pulses were used and the pulse duration at the sample point was ∼100 fs. The layout and other details of the setup in different configurations of the measurements are provided in Figures S2, S3, and S4. Figure S2 shows the configuration 1 of the setup, which was used for determining the optimal thicknesses of the FM and NM layers in their heterostructures for the best THz power generation efficiency. The laser beam is divided into two-part in a 90:10 beam splitter: the stronger part (pulse energy (fluence) of ∼0.35 mJ (1.2 mJ/cm^2^) and beam diameter ∼6mm) is used to pump the THz emitter (spintronic heterostructure), while the weaker part (pulse energy ∼0.4 μJ) was routed through a linear translational stage (for generating computer-controlled time-delays relative to the pump/excitation pulse) and used as the gating beam for the detection of the THz pulse on a nonlinear crystal. The diameter of the gating beam was kept at ∼2 mm using an iris. The collimated pump beam excites the spintronic emitter as shown in Figure S2. The excitation pulse energy or power is controlled by using a neutral density filter. THz radiation emanates from the extended point-like source in the spintronic structure in all possible directions, part of which is collected in the forward direction by using a 15 cm focal length 90^0^ off-axis gold-coated parabolic mirror (OPM1). There are 3 more same-type of OPMs used for giving flexibility to the setup for different experiments (Kumar et al., 2021b, 2021c). After routing the THz beam through the four OPMs, it is focused onto a (110)-surface oriented 0.5 mm thick ZnTe crystal, where, it collinearly matches with the gating pulse in space and time. A high resistive silicon (HR-Si) wafer is placed just before OPM1 to avoid the residual 800 nm pump laser beam completely from reaching the other side of the setup. Commercial neodymium magnets were used to produce a uniform and static magnetic field (B_ext_) of strength ∼120 mT. Detection of the THz pulses takes place on the ZnTe crystal via electro-optic sampling of the gating pulse (Kumar et al., 2021b, 2021c) and achieved using a quarter-wave plate, Wollaston prism, a balanced photodiode, and lock-in amplifier. The pump beam was modulated at a frequency of 267 Hz by an optical chopper.

For quantification of the THz power from the spintronic emitters, we developed a dual-color air plasma-based THz source by reconfiguring the existing setup discussed in Figure S2. The layout of the setup in this configuration (configuration 2) is shown in Figure S3. Of course, in this case, only the THz generation part is modified, while all other optical conditions with the pulse duration, optical alignment, and detection by electro-optic sampling are left unchanged. Therefore, while estimating the THz pulse energy/power from our air-plasma source by comparing it with the reported data in the literature for similar amplified laser pulse duration and pulse energies, any differences can be related to the optical conditioning in the two experiments. For the generation of intense THz pulses from our dual-color air-plasma source, we used a biconvex lens of focal length 15 cm to focus the fundamental and second harmonic beam in the air. Thus, generated white plasma creates forward propagating THz pulses if the fundamental and the second harmonic beams are mixed together properly by adjusting the orientation and crystal angle. We used a β-barium borate (BBO) type-I SHG crystal of thickness 100 μm for generating the second harmonic beam as shown in Figure S3. Thus, emitted THz radiation is collected by the OPM1 and then detected in the ZnTe crystal by electro-optic sampling as describe before. It may be noted from the 4f-geometry of the configurations 1 and 2 in Figures S2, S3, and S4 that the difference in the size of the THz source in the two (the optically excited region within the spintronic heterostructure and the air plasma, respectively) will result into a difference in the size of the THz beam on the nonlinear crystal for its detection. The optical gating beam size on the nonlinear crystal was kept the same at ∼2mm. Therefore, it will amount to an underestimation of the actual THz power from the spintronic source, if no change in the position of the nonlinear crystal and other optical alignments are made in the setup. The excitation pulse energy-dependent THz output of our air-plasma source was compared with that from the literature for a similar setup and optical conditions with the excitation pulse and detection.

For doing excitation power-dependent measurements on the spintronic heterostructures for evaluating their THz generation efficiency, we modified the setup to configuration 3 as shown in Figure S4. Here, the spintronic source is placed between the two inner parabolic mirrors and irradiated with a converging excitation beam allowed through the aperture in the second OPM, and excitation power was controlled by using a neutral density filter. The diameter of the optical beam on the sample was ∼1 mm. Like before, no change in the optical conditioning with all other things including the electro-optic detection, we made. Therefore, comparisons among different experiments for relative performance parameter was possible. Again, a HR-Si wafer was placed just after the emitter to avoid the residual pump from reaching the second part of the setup.

## Data Availability

There is no dataset or code associated with this work.
