# Supplementary material for: Optical damage limit of efficient spintronic THz emitters
Source: iScience. 2021 Sep 21;24(10):103152. doi: 10.1016/j.isci.2021.103152 (PMC8496183; doi:10.1016/j.isci.2021.103152)
Supplement: Document S1. Figures S1–S8 [file mmc1.pdf]

iScience, Volume *24*

Supplemental information

Optical damage limit of efficient spintronic THz emitters

Sandeep Kumar, Anand Nivedan, Arvind Singh, Yogesh Kumar, Purnima Malhotra, Marc Tondusson, Eric Freysz, and Sunil Kumar

Supplimental Information

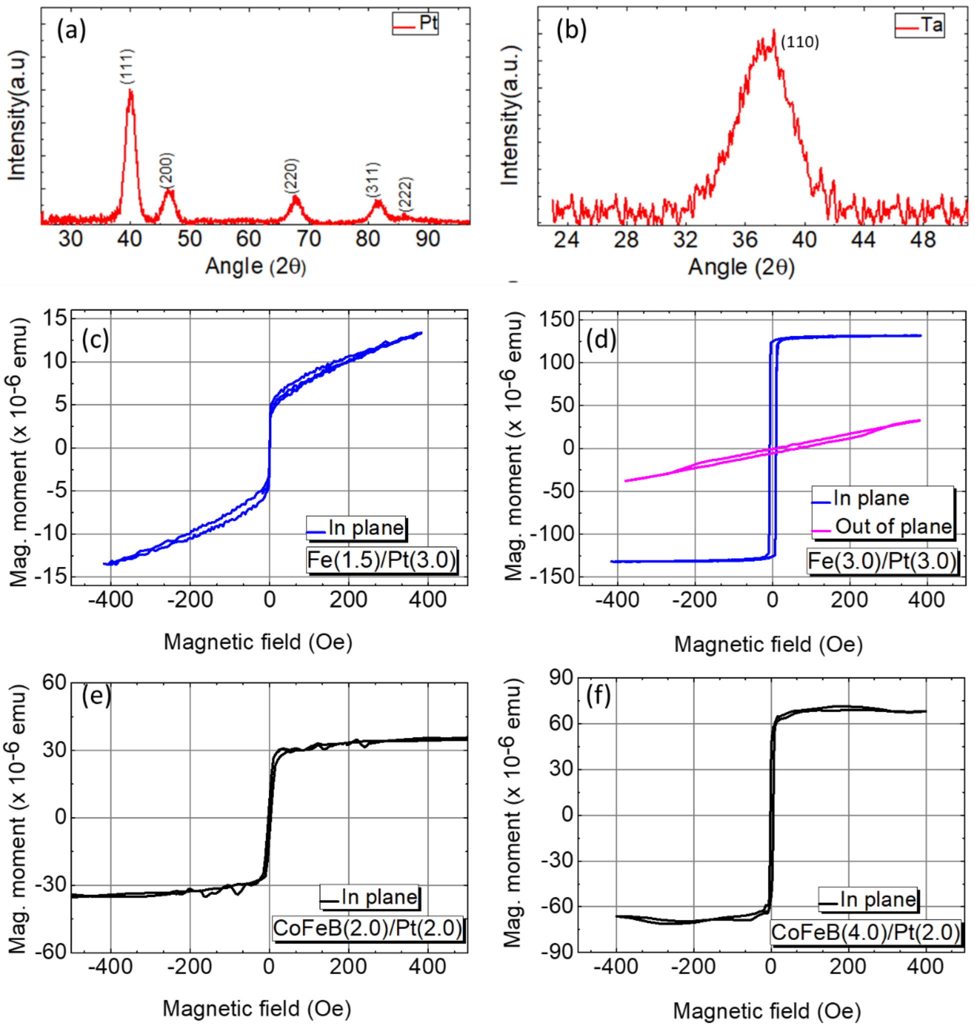

Figure S1 XRD plots of (a) Pt film, and (b) α-phase Ta film. The crystallographic planes corresponding to various XRD peaks have been marked. Hysteresis loops from in-plane and out-of-plane magnetic measurements using vibrating sample magnetometer on (c) Fe(1.5)/Pt(3.0), (d) Fe(3.0)/Pt(3.0), (e) CoFeB(2.0)/Pt(2.0), and (f) CoFeB(4.0/Pt(2.0) bilayer FM/NM spintronic heterostructures. Related to **Star Method.**

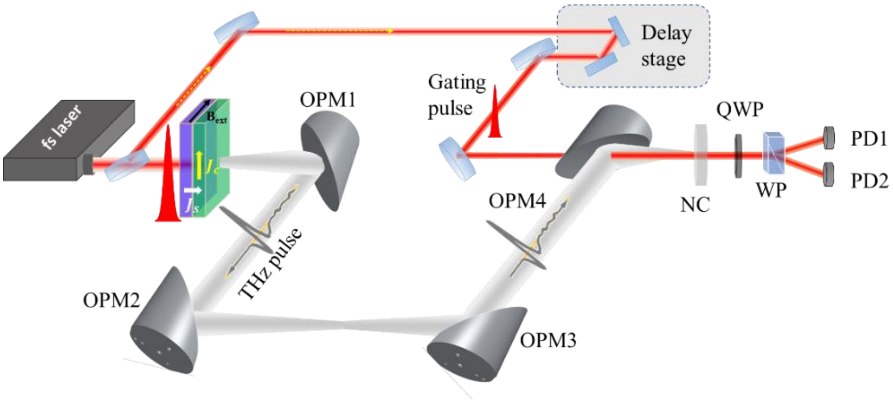

Figure S2 Configuration 1: Time-domain THz spectroscopy setup using spintronic emitters. Pulsed THz generation from the excitation of the spintronics heterostructures with femtosecond NIR pulse and probing of the THz pulses by electro-optic sampling in a nonlinear optical crystal. OPMs: off-axis parabolic mirrors, QWP: quarter-wave plate, NC: nonlinear optical crystal, WP: Wollaston prism, PD: photodiode, LIA: lock-in amplifier, B_{ext}: applied external magnetic field, J_s: spin current, J_c: charge current. Related to **Star Method**.

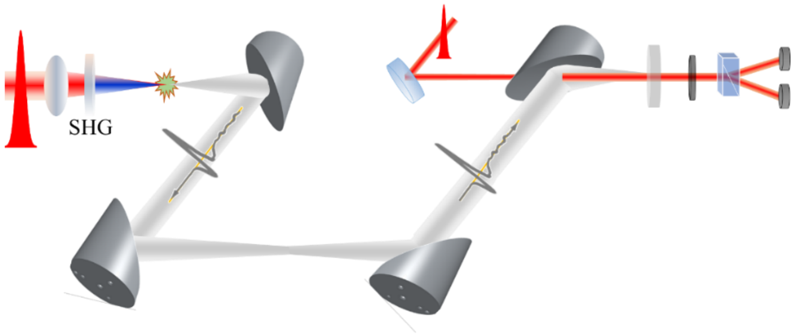

Figure S3 Configuration 2: Time-domain THz spectroscopy setup using dual-color air-plasma source. SHG: second harmonic generation crystal. Related to **Star Method.**

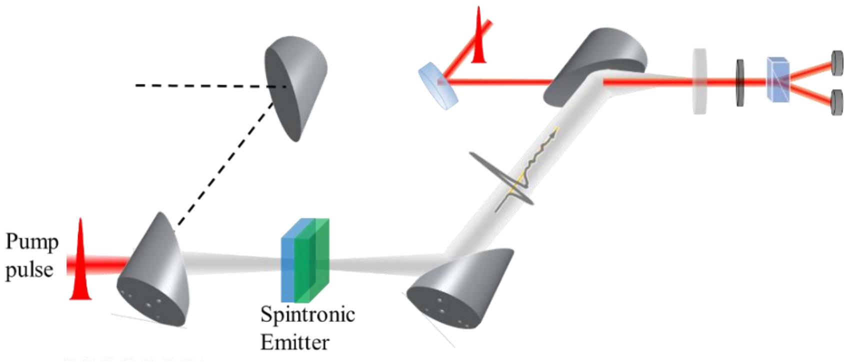

Figure S4 Configuration 3: Experimental setup for THz time-domain spectroscopy using spintronic emitters and to determine their optical damage threshold. Related to **Star Method.**

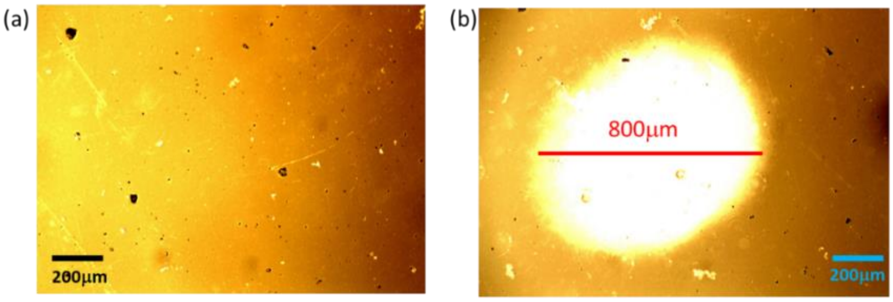

Figure S5 Optical microscopy images of the representative spintronic emitter taken in the transmission mode using a 10X objective lens, (a) before and (b) after the optical damage. Related to **Figure 7**.

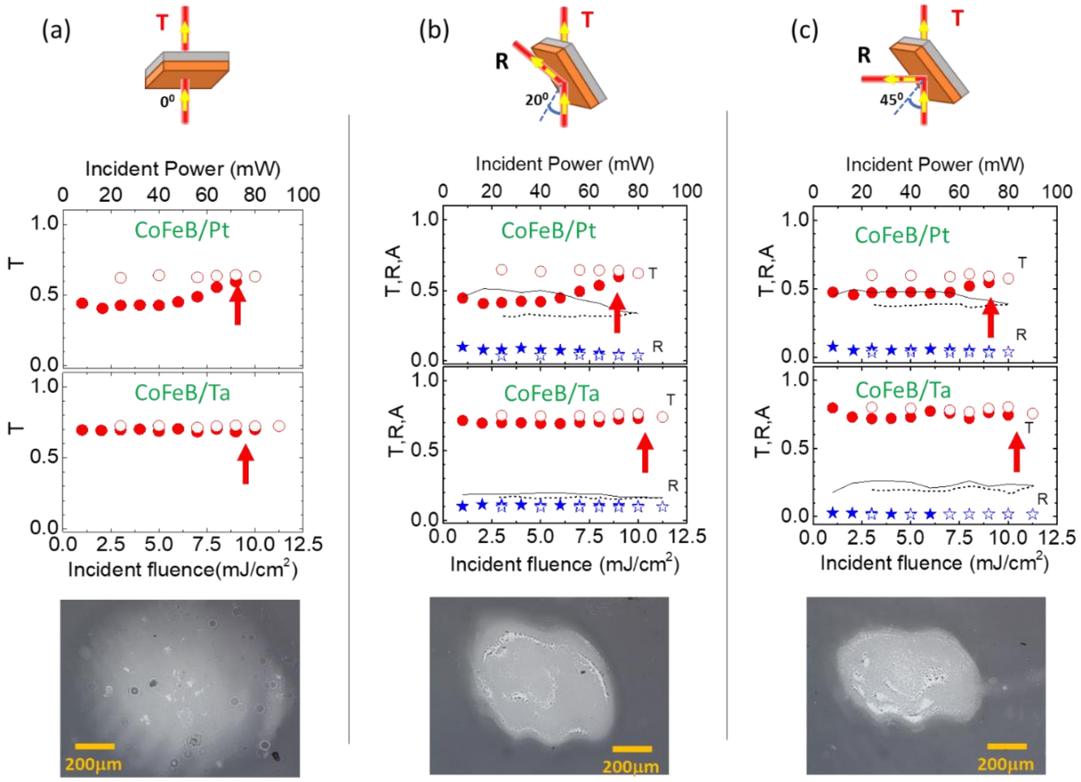

Figure S6 Optical transmittance (T) and reflectance (R) measurements with respect to the incident laser fluence (power) on the spintronic emitters at three different incident angles as indicated. The representative samples are CoFeB(3)/Pt(3) and CoFeB(3)/Ta(3), both on quartz substrates. Solid symbols are for increasing power while the open symbols are for lowering power values. The optical microscopy images were taken after the optical damage of the CoFeB/Pt sample at certain fluences (powers) indicated by vertically up arrows. There was hardly any damage to the CoFeB/Ta sample on the quartz substrate upto the highest experimental fluence. Thin continuous and dotted black lines in (b) and (c) are the absorptance values while increasing and decreasing the excitation fluence, respectively. Related to **Figure 8**.

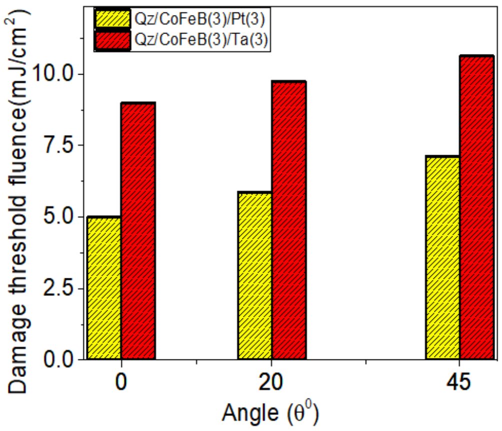

Figure S7 Angle of incidence dependent variation in the damage threshold fluence for CoFeB(3nm)/Pt(3nm) and CoFeB(3nm)/Ta(3nm) bilayer heterostructures on quartz substrate. Related to **Figure 8**.

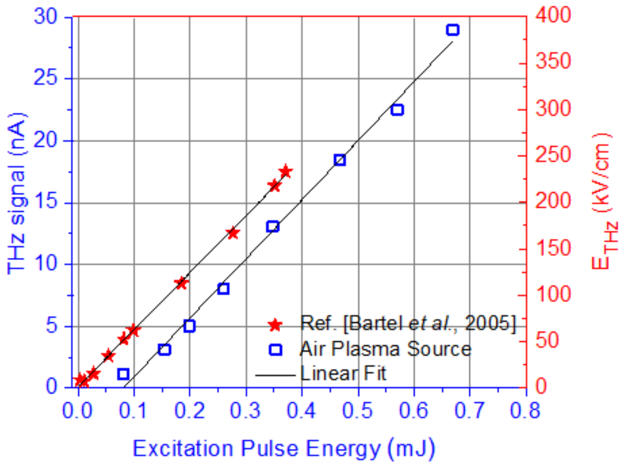

Figure S8 Comparison in THz signal generation from the coherent air-plasma source in our experiments and the literature [Bartel *et al.*, 2005]. At any given excitation pulse energy, the THz field is measured in nA in our experiments and in kV/cm in the literature [Bartel *et al.*, 2005]. The solid line represents the linear dependence of the THz field strength on the excitation pulse energy. Related to **Figure 6.**
